# TapFix: Cursorless Typographical Error Correction for Touch-Sensor Displays

**DOI:** 10.3390/s25051421

**Published:** 2025-02-26

**Authors:** Nicholas Dehnen, I. Scott MacKenzie, Aijun An

**Affiliations:** Department of Electrical Engineering and Computer Science, York University, Toronto, ON M3J 1P3, Canada; nilexad@gmail.com (N.D.); aan@yorku.ca (A.A.)

**Keywords:** mobile phone, text entry, text correction, touchscreen, cursorless, typing error

## Abstract

We present TapFix, a cursorless mobile text correction method for touch-sensor displays. Unlike traditional methods, TapFix eliminates the need to position a cursor to make corrections. Instead, the method allows for direct, character-level access, offering easy-to-use swipe gestures on a zoomed-in target word for corrective actions. A user study with 15 participants compared TapFix to two traditional text correction methods on Apple iOS. For each of the three methods, participants completed 100 text correction tasks of four different error types on an Apple iPhone 14 Pro. The TapFix method was on average between 43.0% and 44.1% faster than the existing methods in completing the tasks. Participants also reported experiencing 5.6% to 21.1% lower levels of frustration with TapFix, as indicated by post-experiment NASA TLX and SUS questionnaires, compared to the traditional methods. Additionally, they attributed a level of usability to TapFix that was comparable to the well-established TextMagnifier method.

## 1. Introduction

**Figure 1:**
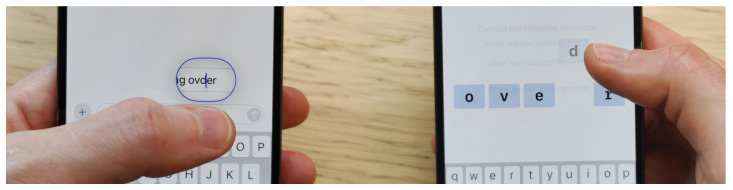
A user correcting a mistyped word on the touch-sensing display using an Apple iPhone with TextMagnifier (**left**) and TapFix (**right**).

In the modern world, mobile phones are ubiquitous. According to a 2022 ITU annual report, in more than half the countries in the world, over 90% of all people own a smart phone [1]. Young adults in particular spend on average two to five hours a day on their mobile phones [2], a large chunk of which involves text input, such as messaging, social media, or web browsing [3]. Since 2008, mobile phone manufacturers have moved away from physical input methods such as T9 or physical keypads. With the release of the Apple iPhone, capacitive touch-sensor screens became increasingly popular, as touch interaction enabled natural and direct access to objects of interest, thus saving time and increasing productivity [4]. While touch-sensor screen usability has steadily improved with the introduction of higher resolution and multi-touch displays, one major drawback that cannot be solved by improvements in hardware is occlusion [5,6]. When interacting with a mobile device, a large portion of the small screen is occluded by the comparably “fat finger” [7]. This is seen in Figure 1 (left) where the thumb occludes almost all of the text during selection, creating uncertainty as to the exact spot on the screen where the input is registered. This uncertainty is especially impeding for text input, as the user has to precisely select the letters on a small soft keyboard, which in turn leads to errors. Attempts to solve this problem include techniques such as improving touch-sensor accuracy by shifting touch events [8], using individualized machine learning methods [9,10], or accessing other sensor data to improve movement [11].

Text-based methods contrastingly work at the character or word level to suggest potential next characters or to correct typing mistakes. Autocomplete offers word suggestions after the first few letters typed to reduce the keystrokes for a word or sentence [12]. Autocorrect on the other hand provides suggestions for mistyped words but requires a word separation character (such as space or period) [13,14]. These methods are not without disadvantages: While Autocomplete reduces the number of characters to type a word, the user must attend to, consider, and click on potential suggestions. This negatively impacts typing speed [15,16]. In addition, although current Autocorrect algorithms are accurate, they often fail to offer sensible correction suggestions for misspelled names and other proper nouns, or even worse, actively change correct words into an assumed, corrected counterpart, requiring the user to manually intervene [17]. This mainly affects non-western names and nouns (e.g., correcting “Shinobu” into “Shining”), which led to campaigns against discrimination in Autocorrect, such as “Elimn8Hate” (https://www.namereclaim.ca; accessed on 23 February 2025) and “I Am Not a Typo” (https://www.iamnotatypo.org; accessed on 23 February 2025), the latter of which was founded by journalist Dhruti Shah and law professor Rashmi Dyal-Chand, who regularly found their names corrected into Dorito and Sashimi, respectively [18]. English sociolects, such as the African American Vernacular English (AAVE), and the many popular modern slang terms borrowed from them, are also affected [19,20].

To manually correct a typo, or typographical error, the user must position the cursor between letters, a task even more affected by occlusion than typing itself, as the target area is smaller.

**Figure 2:**
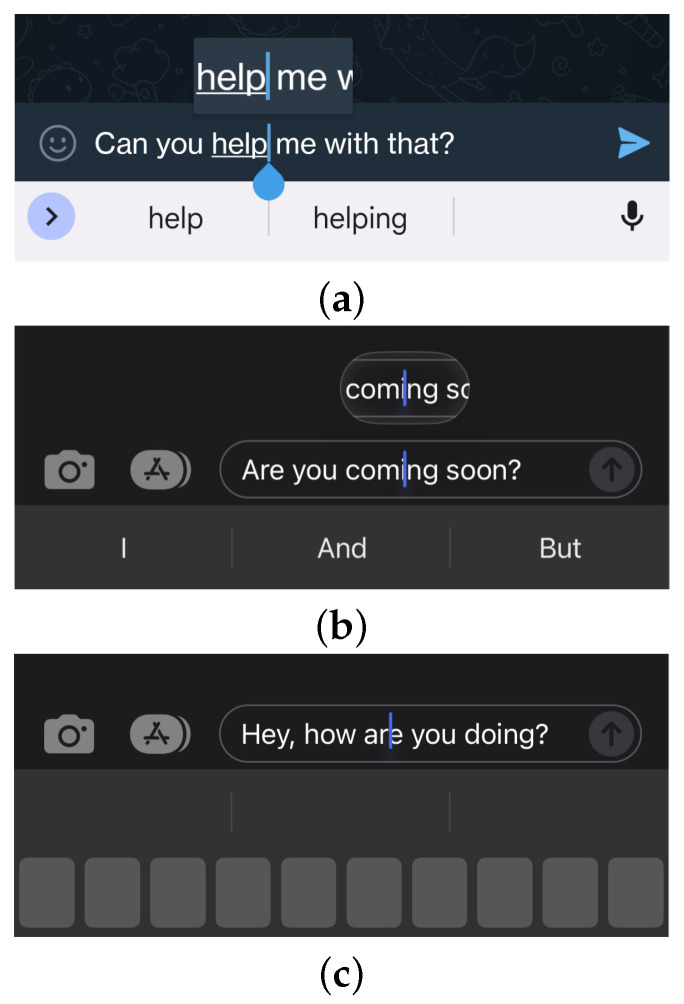
Different cursor positioning methods on touch-sensing displays. (**a**) Cursor positioning with magnifier and handle on Android; (**b**) Textfield long-press cursor positioning with magnifier on iOS; (**c**) Cursor positioning through horizontal swiping on space-bar with automatically greyed out keyboard on iOS.

Manufacturers have come up with different solutions for this problem, three of which are seen in Figure 2. For example, Google’s Android keyboard offers a tear-shaped handle under or above the cursor (Figure 2a), to increase the target area for “grabbing” the cursor and to solve the occlusion issue. Apple provides two methods for cursor positioning. The first (Figure 2b) involves long pressing and holding a finger down on the text, making a zoomed-in version of the cursor and text appear above the finger, which can then be slid around to find the correct position. The second (Figure 2c) allows the user to long press on the space bar, which in turn blacks out all keys and allows the user to slide her finger left and right on the keyboard area to position the cursor accordingly.

These methods, however, are awkward and slow down text input on mobile devices with touch-sensing displays. In this study, we propose TapFix. As seen in Figure 1 (right), TapFix is cursorless and side-steps the occlusion problem noted earlier. We evaluate TapFix against existing cursor positioning methods.

The following section reviews related research. Section 3 presents TapFix, a novel correction method for character-level replacements during mobile text entry. Then, in Section 4, our methodology is explained. Finally, in Section 5, results are presented and discussed.

## 2. Related Work

Although there exists a lot of research on implementations and visualizations of Autocomplete and Autocorrect, little exists outside of this spectrum, e.g., on general cursor placement or manually correcting mistakes when Autocorrect fails. Three papers are particularly relevant. See Table 1.

Zhang et al. [21] present three methods to circumvent the issue of cursor placement. They allow the user to type a correction, then either drag it onto the erroneous word (Drag-n-Drop), flick it in the direction of the word (Drag-n-Throw), or use a Magic Key. Magic Key automatically highlights preceding words using a neural network to choose words most likely to be typos and allows the user to immediately apply the correction. The authors found a statistically significant effect of correction method on the average text correction time, with their proposed methods up to 1.8 s faster than the traditional cursor-based method. The only outlier was Drag-n-Drop, which slowed down text insertions beyond the average speed of the traditional method. Furthermore, when asked to rate usability on a System Usability Scale (SUS) questionnaire [24] and workload on the NASA Task Load Index (TLX) [25], participants showed a slight preference for the three new methods over the traditional method. However, success rates were 88% to 97%, leaving these methods in the range of traditional Autocorrect [26].

Cui et al. [22] improved on the results from Zhang et al. with a correction method coined JustCorrect. Their method is the same as Magic Key, except the magic key immediately commits the correction entered by the user. To achieve this, they use an algorithm which ranks replacement candidates based on multiple scores (Levenshtein distance, semantic similarity, and an *n*-gram language model). It then replaces the most likely candidate. They report an improvement of text correction time of 12.8% over the traditional Android keyboard and 9.7% over the techniques presented by Zhang et al. [21].

Lastly, Swap by Li et al. [23] employs a similar technique to Magic Key. Instead of using algorithmic highlighting or computation, users decide where and what to insert, delete, or replace. One difference to Magic Key is the use of a modified text layout in correction mode, with large empty boxes between words to simplify insertion of text. They found a significant effect of correction method on error correction time, with Swap having the shortest correction time on average.

## 3. TapFix

**Figure 3:**
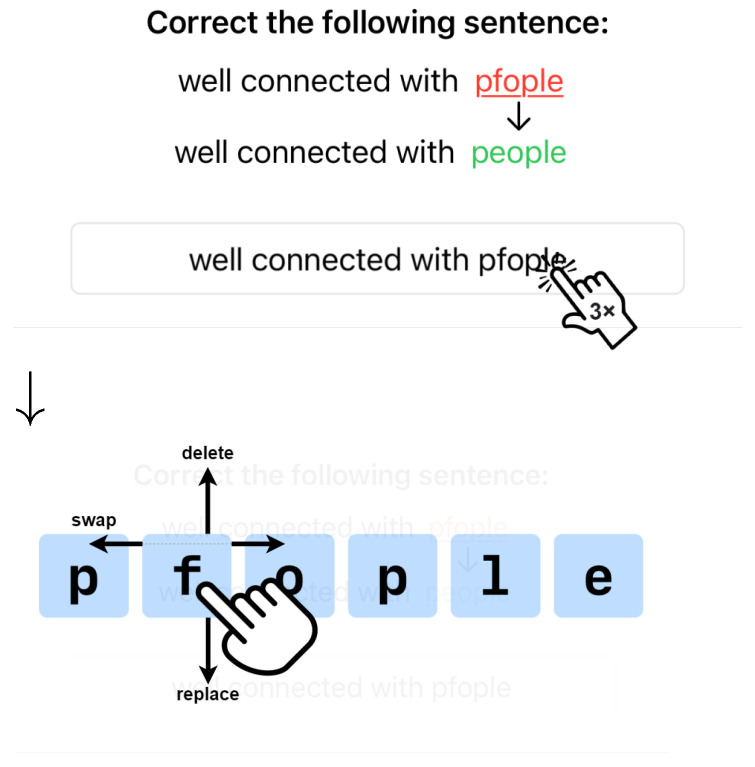
The TapFix correction method: After entering the text correction view with a triple tap, the user has direct access to character buttons.

The method proposed herein operates at a character level and allows for quick insert, delete, replace, or swap with taps and swipes. Figure 3 shows TapFix’s text correction flow, implemented in a custom application for iOS.

To enter correction mode, a triple-tap is used. The choice for a triple-tap was pragmatic as double-tap or touch-and-hold conflict with existing operating system gestures. A commercial implementation of TapFix could exploit context and use a double-tap or touch-and-hold gesture. After triple tapping on a word, the user interface is greyed out, and a large representation of the word appears in the center of the screen. The keyboard is opened at the same time, if it was not already visible to the user. Each letter is shown as a separate button for the user to directly interact with. Five interactions are supported:

1. Drag letter up ⇒ delete a letter. This is visualized to the user by a decreasing opacity of the letter the farther it is dragged up (shown in Figure 1, right).
2. Drag letter down ⇒ activate replacement mode. The next character entered replaces the character selected. This is visualized as a change in color of the letter; once replacement mode is active, the targeted letter is highlighted in red.
3. Press key on keyboard ⇒ insert a letter. The corresponding character is inserted in the middle of the word displayed. The character can then be moved to the correct position via drag and drop. Insertions require the replacement mode to be inactive.
4. Drag and drop ⇒ swap mode. Touch and hold a character and move it left or right to reposition it within the word.
5. Tap elsewhere on screen ⇒ exit correction mode.

**Figure 4:**
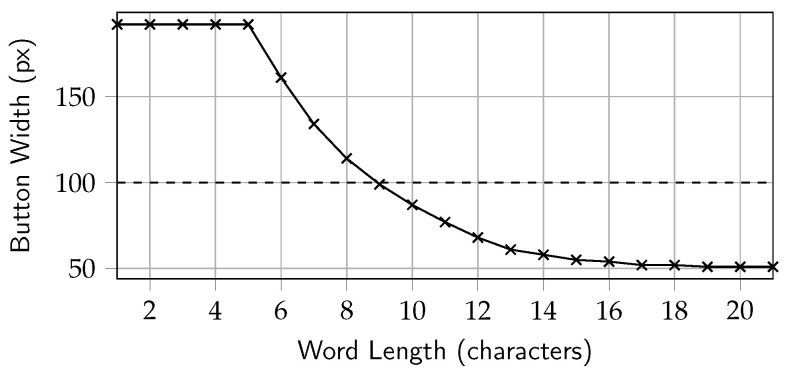
Width scaling of TapFix character buttons.

Depending on the size of the word to correct, the character buttons in TapFix automatically adjust their size to fit the screen. Starting at a width of 64 pt and a height of 50 pt (points) for words up to 8 characters, buttons decrease in width down to 17 pt at 21 characters in order to still fit on the screen. On Retina devices, the number of pixels depends on the scale factor. For all iPhones released since 2020 (with the exception of the SE models), this factor is 3.0, thus 1 pt will scale by 3 px (pixel) into each dimension. The default character button width on the English (US) keyboard is 100 px. So, for up to 9 characters inclusive, TapFix characters will be at least as wide as a regular keyboard character. The scaling curve for the width of TapFix character buttons is shown in Figure 4. The effect of the character button width and the target word length in general is investigated as part of the experiment.

TapFix was developed with a focus on English text entry. However, the method is generally language- and keyboard-agnostic and could (with adjustments) function with alternative input methods, such as hand-writing characters or the Chinese Wubihua method Also commonly referred to as the “Stroke Count Method”. The only requirement is that the language’s writing system allows for words to be broken into individual characters.

## 4. Method

In this paper, an experimental methodology was employed. In a user study, TapFix was empirically evaluated and compared against the existing text correction methods on iOS (described in Section 1 and shown in Figure 2). The hypothesis is that due to the absence of cursor positioning to make text corrections and direct character-level access, the proposed method will outperform the existing methods in terms of error correction speed.

### 4.1. Participants

Fifteen computer-literate, adult participants were recruited on the local university campus. Three participants owned Android-based cell phones, while the others used iPhones. Regardless of their phone’s operating system, all participants indicated familiarity with the TextMagnifier method for cursor positioning. Participants were predominantly between 25 and 34 years old and stated they used their mobile phones 3–4 h per day. For participating in the study, participants were offered a compensation equivalent in value to US $15.

### 4.2. Apparatus

The experiment was conducted on the touch-sensing display on an Apple iPhone 14 Pro running iOS 17.3 (Apple Inc., Cupertino, CA, USA). The phone is 147.5 mm tall and 71.5 mm wide and has a screen resolution of 1179 by 2556 pixels. All text input assistance functionality on the device (such as Autocorrect or Autocomplete) was disabled during testing. To present the experiment tasks, a custom testing application was written in Swift and installed on the device.

The software guided participants through the testing using the TapFix and measured user performance. The application source code is available on GitHub (https://github.com/nicholasdehnen/tapfix-userstudy-ios (accessed 23 February 2025)). Additionally, the questionnaires were presented in paper form.

### 4.3. Procedure

Participants sat at a table during day time. The software and experiment was explained and demonstrated to the participants.

**Figure 5:**
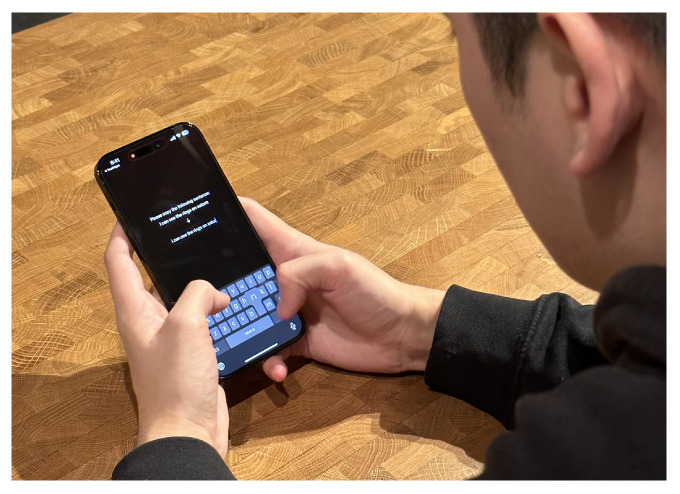
A participant working on a typing warm-up task.

All participants held the device with both hands and used their thumb(s) to interact with the device and type on the keyboard. Figure 5 shows a participant performing the typing warm-up task.

No quantitative data was recorded on how participants held their phones or potentially changed their grip during the trials. However, most participants used the two-thumb method for the writing warm-up and switched to the one-handed (as shown in Figure 1 for example), using solely the thumb of their dominant hand to correct the typos.

#### 4.3.1. Typing Warm-Up

A warm-up typing round was performed to ensure an even level of familiarity with the testing device and its text input functionality among the participants. As most participants owned different models of the device itself, this allowed them to adjust to the screen size of the testing device.

**Figure 6:**
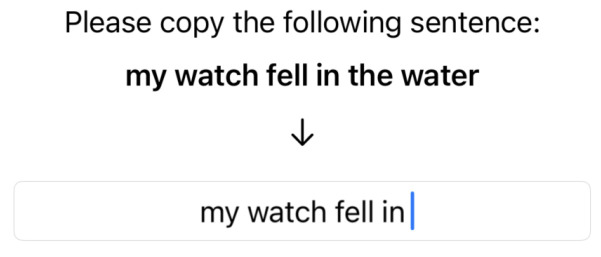
Typing warm-up prompt.

Autocomplete and Autocorrect were programmatically disabled during the typing warm-up. Additionally, participants were not allowed to correct any typing mistakes they made. A total of 20 sentences were randomly selected from the MacKenzie and Soukoreff phrase set [27], which participants had to copy. A screenshot of this warm-up process is depicted in Figure 6 and is also seen on the device in Figure 5.

#### 4.3.2. Correction Warm-Up

**Figure 7:**
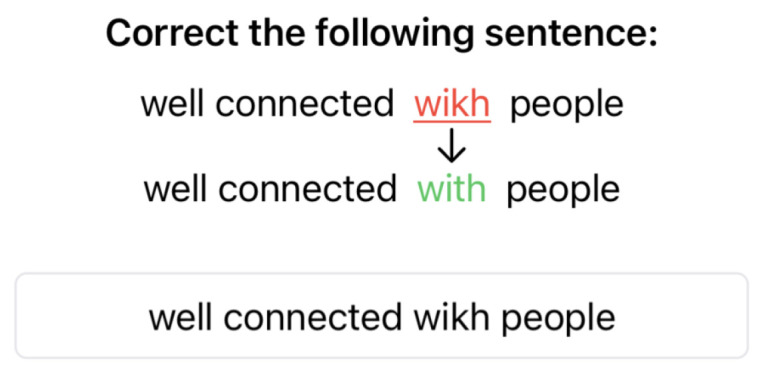
Visualization of the induced error for the user to correct.

Preceding each correction test, warm-up rounds for the corresponding method were performed, one per correction type. Correction types included deletion of an added character, replacement of a character, insertion of a missing character, and swapping the order of two characters. Each round included 10 randomly selected sentences from the phrase set mentioned above. Depending on the correction type, an error was introduced at a random location in a word by randomly replacing a letter (Replace), inserting an additional character (Delete), removing an existing character (Insert), or swapping two consecutive characters (Swap). Special care was taken to ensure there would be no ambiguity in the resulting “mistyped” word. The error was visualized to participants above the text input box as shown in Figure 7.

The text input box was pre-populated with the erroneous sentence, in order to mimic a situation where a user checks their typed sentence, for example, before sending it on a messaging app. The time measurement started once the user touched the text box and finished once the error was corrected.

#### 4.3.3. Trials

Each participant then performed three trials per text correction method and correction type, administered in a randomized order. Each test was comprised of 25 corrections. Erroneous sentences were generated in the same fashion as described in Section 4.3.2. The current correction method and type of the tasks were explained to participants in a screen preceding the actual tests. Additionally, it was displayed at the upper right corner of the screen.

Measurement of error correction time started as soon as the participant touched the text field. If a participant failed to correct an error—for example, due to accidentally replacing or deleting a different character—the trial was marked as invalid. For every invalid trial, another trial was appended to compensate. Where possible, the testing application automatically caught these mistakes; however, not all user errors are easily detectable in software. Therefore, participants were additionally given the option to manually mark trials as invalid using a “Flag” button in the upper right corner of the testing app. Prior to testing, participants were encouraged to use this flagging functionality to mark trials whenever they felt they did something wrong.

#### 4.3.4. Questionnaires

After completion of all trials, participants were asked to provide qualitative feedback on the correction methods using the System Usability Scale [24] (see Appendix A.1.1), as well as rating the workloads using the NASA-TLX [25] (see Appendix A.1.2). The questionnaires were administered in a paper-based format and included additional space for participants to give free-form comments on each method, if desired.

### 4.4. Design

The experiment was a 3×4 within-subjects design. The independent variables and levels were as follows:

- Correction method (SpacebarSwipe, TextMagnifier, TapFix);
- Correction type (Insert, Delete, Replace, Swap).

The dependent variables were task completion time (s) and cursor positioning time (s). The latter time was measured as the time for participants to touch the erroneous character (TapFix) or to place the cursor behind the character to be corrected (SpacebarSwipe and TextMagnifier).

The total number of trials per participant was 3×4×25=300, resulting in 15×300=4500 trials in total.

## 5. Results & Discussion

The majority of the trials were completed successfully. In one case, a software bug occurred during a task involving the TextMagnifier method, which lead to an invalid task completion time being recorded. This result was manually removed upon review of the test data. Besides that, user errors lead to a number of tests being marked invalid. For example, a common issue with SpacebarSwipe was that any movement prior to the activation of the method (through long pressing the space bar) led to cancellation of the activation, in some cases also followed by the accidental insertion of a letter. In total, 356 out of 4856 trials (7.3%) were marked as invalid, either through automated means, or by the participants themselves. Each participant therefore performed about 24 additional trials on top of the 300 trials initially scheduled.

The data from the experiment were processed in Python (version 3.10.8) using Pandas (version 1.5.3), NumPy (version 1.24.1), and Seaborn (version 0.12.2) to perform data aggregation, calculate averages of the various measures and generate charts. Statistical analysis was performed using the Python Pingouin library (version 0.5.4), and the results were cross-checked with GoStats (https://www.yorku.ca/mack/GoStats/; accessed on 23 February 2025) application.

### 5.1. Text Entry Speed

**Figure 8:**
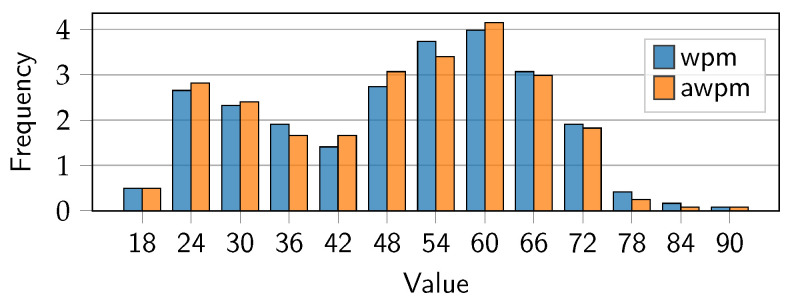
Warm-up trials. Distribution of text entry speed in words per minute (wpm) and adjusted words per minute (awpm) over frequency of sentences typed.

For the warm-up trials, despite not correcting mistakes, participants achieved an average accuracy of 98.2% (*SD* = 2.75) over all 20 trials, with a minimum of 83.3% and a maximum of 100.0%. These accuracy numbers were calculated using the Levenshtein minimum string distance between the typed string and the correct string [28]. Participants took on average 7.88 s (*SD* = 3.65) to type a sentence. Considering the average sentence length to be 28.1 characters (*SD* = 3.67), this resulted in an average typing speed of 49.5 wpm (*SD* = 16.18), calculated according to MacKenzie [29], and 49.5×0.982=48.6 wpm adjusted for accuracy (awpm). The distribution of typing speeds is shown in Figure 8.

### 5.2. Task Completion Time

**Figure 9:**
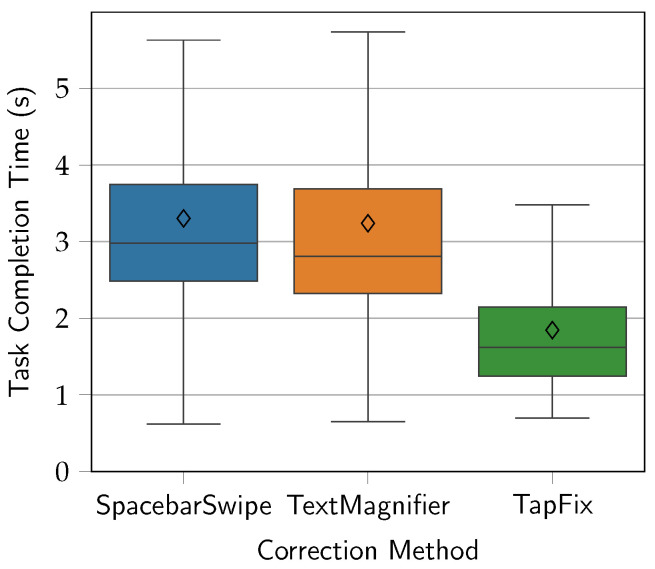
Task completion time (s) by correction method, combining all four correction types. Diamonds show the mean times.

The grand mean for task completion time was 2.80 s. The mean times per correction method were 3.30 s for SpacebarSwipe, 3.24 s for TextMagnifier, and 1.85 s for TapFix. As such, TextMagnifier trials were 1.96% faster than SpacebarSwipe trials, and TapFix trials were 79.0% faster than SpacebarSwipe trials and 75.6% faster than TextMagnifier trials. The distribution of the results, as well as minimum and maximum values, is seen in Figure 9. Note that data points above the upper fence are omitted in order to prevent distortion and retain readability of the plot.

A two-way repeated-measures analysis of variance (ANOVA) was conducted to compare the effect of the correction method and type on the task completion time. Shapiro–Wilk tests and a manual inspection of the Q–Q plot confirmed a satisfactory level of normality across all conditions. Mauchly’s sphericity test was conducted to assess the assumption of sphericity. The test indicated that the assumption of sphericity was not violated ($\chi^{2}=80.2$, p=0.18).

**Figure 10:**
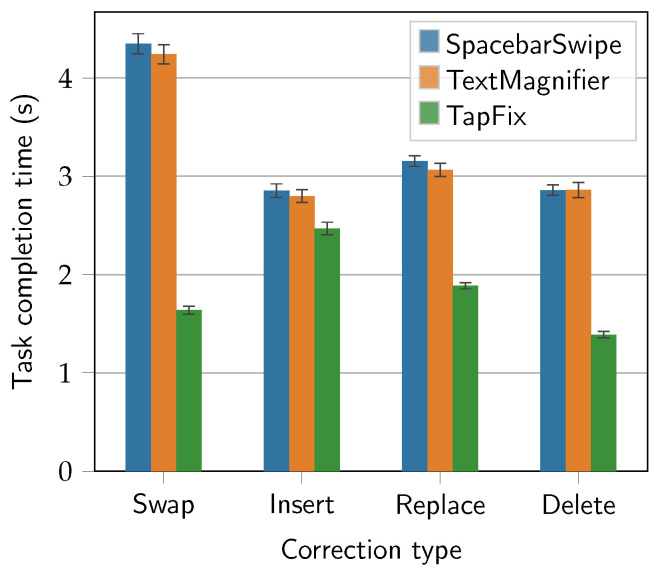
Task completion time (s) by correction type. Error bars show ±1 *SE*.

The effect of the correction method on the task completion time was statistically significant ($F_{2,28}=138.4$, p<0.0001). The effect size indicated a large effect ($\eta_{p}^{2}=0.91$). Figure 10 shows the data points grouped by correction type. Furthermore, the effect of the correction type on the task completion time was statistically significant ($F_{3,42}=22.2$, p<0.0001), and the effect size indicated a large effect ($\eta_{p}^{2}=0.61$) as well. This effect, however, was expected and is not further relevant to the comparison at hand.

The CorrectionMethod × CorrectionType interaction effect was statistically significant ($F_{6,84}=24.1$, p<0.0001). The effect size again indicated a large effect ($\eta_{p}^{2}=0.63$). A post hoc analysis using pairwise *t*-tests revealed that the task completion time was significantly different for correction types Delete, Replace, and Swap, for each between the pairs of SpacebarSwipe and TapFix, as well as TapFix and TextMagnifier. Bonferroni corrections were applied to adjust for multiple comparisons. Task completion time for any correction type task between methods SpacebarSwipe and TextMagnifier was in no case significantly different. For correction task type Insert, there was no significant difference between pairs of any of the three methods. All *t*-test results, including the *t*-statistics, uncorrected ($p_{unc}$) and Bonferroni-corrected ($p_{cor}$) p-values, and significance indication are found in Table 2. Note that for the sake of brevity, method names are abbreviated.

A large difference between the Swap-type task results for TapFix (*M* = 1.64 s, *SD* = 0.79) and the two baseline methods, SpacebarSwipe (*M* = 4.35 s, *SD* = 1.98) and TextMagnifier (*M* = 4.24 s, *SD* = 1.88), can be observed. While the baseline methods require multiple Delete and Insert actions to correct an instance of disordered characters, the direct character-level access in TapFix allows for a correction with a single (i.e., for two swapped characters) drag-and-drop action. Post hoc pairwise *t*-tests for the Swap correction type confirmed this, revealing a significant difference between SpacebarSwipe and TapFix ($t_{28}=7.53$, p<0.0001), as well TapFix and TextMagnifier ($t_{28}=-8.58$, p<0.0001). These are promising results for TapFix. The difference between SpacebarSwipe and TextMagnifier was not statistically significant ($t_{28}=0.25$, p>0.05).

Conversely, the results for the Insert correction type only show a minor advantage in task completion time for TapFix (*M* = 2.47 s, *SD* = 1.24) compared to SpacebarSwipe (*M* = 2.85 s, *SD* = 1.34) and TextMagnifier (*M* = 2.80 s, *SD* = 1.25). This difference was not statistically significant, neither for SpacebarSwipe and TapFix ($t_{28}=1.70$, p>0.05), nor for TapFix and TextMagnifier ($t_{28}=-1.59$, p>0.05), nor SpacebarSwipe and TextMagnifier ($t_{28}=0.26$, p>0.05). This may be linked to the behavior of the TapFix insertion functionality, where an inserted character is added in the middle of the word (see Section 3): Three participants noted in their comments regarding the TapFix method that they occasionally lost track of the letter just inserted, especially when there were one or more of the same letter already in the word. This additional cognitive load may have impacted the overall speed during the TapFix-Insert tasks. A potential remedy is highlighting the newly inserted letter: Just like the Replace mode colors the character to be replaced red, the newly inserted character could appear green as a visual aid. Additionally, a different insertion strategy could be employed, such as always inserting the new character at the right-hand side (or left, in case of left-handedness), or exploring the use of machine learning models to find a suitable location for the new character.

Finally, an entirely different insertion mechanism may be considered. While the current TapFix insertion flow requires two actions, inserting followed by dragging the character to the correct position, this could be reduced to a single one by allowing drag-and-drop directly from the keyboard into the word. Presumably, this would lead to a similar decrease in task completion time as seen with the remaining three correction types. However, from an implementation point of view this would be a challenging option, requiring the implementation of a custom keyboard.

The Delete and Replace correction tasks exhibited the same outcome as Swap, though in a less extreme fashion. This effect was almost certainly caused by the difference in the number of required corrective actions: 1 to 2 for Replace and Delete and 4 for Swap. Compared to SpacebarSwipe and TextMagnifier, TapFix was respectively 51.4% faster than both on the Delete task and 37.6% (SpacebarSwipe) to 40.2% (TextMagnifier) faster on the Replace task. This difference was statistically significant, as shown by pairwise *t*-tests: For Delete, pairs SpacebarSwipe and TapFix ($t_{28}=9.68$, p<0.0001) and TapFix and TextMagnifier ($t_{28}=-6.64$, p<0.0001) differed significantly. The same result was obtained for Replace where the pairs SpacebarSwipe and TapFix ($t_{28}=7.55$, p<0.0001) and TapFix and TextMagnifier ($t_{28}=-6.38$, p<0.0001) also differed significantly. The difference between SpacebarSwipe and TextMagnifier was not statistically significant for Delete ($t_{28}=-0.00$, p>0.05) or for Replace ($t_{28}=0.47$, p>0.05).

As all three methods require only a single corrective action for the Delete task, this is an interesting metric to examine. The difference in task completion times for this correction type is therefore solely due to the differences in time to activate the method and position the cursor. TapFix seems to have an advantage here, requiring less than half the time of the baseline methods to achieve this. Further analysis of the composition of each methods task completion time will be done in the following section.

#### 5.2.1. Composition

**Figure 11:**
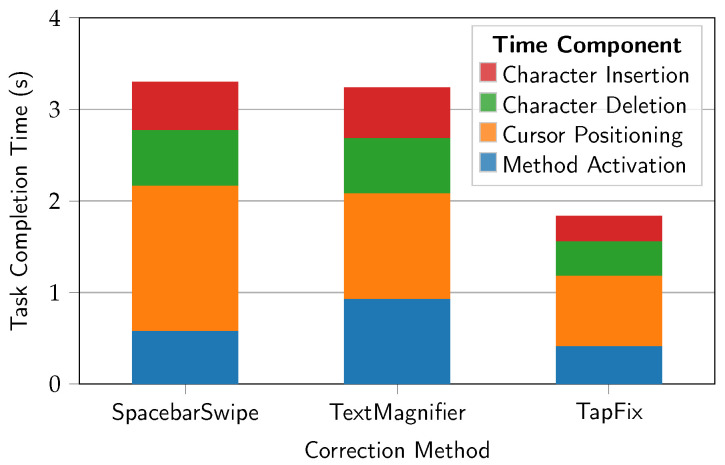
Task completion time composition (s).

Figure 11 shows the task completion time, broken down by time required to activate the method, position the cursor, and finish the insertion or deletion (or both, for Swap).

The mean method activation time for TapFix was 0.41 s (*SD* = 0.34). This was measured from the first touch to the completion of the triple-tap gesture. For SpacebarSwipe, the mean activation time was 0.58 s (*SD* = 0.02) and for TextMagnifier 0.93 s (*SD* = 0.04). Note that the standard deviation for the baseline methods is much lower, as the method activation time is likely hard-coded in the OS and starts from the moment the user touches the screen, whereas a sequence of three taps allows for more variability. Pairwise comparisons using Welch’s unequal variances *t*-test revealed a statistical significance of the difference in activation times, for SpacebarSwipe and TapFix ($t_{11.11}=12.09$, p<0.0001), SpacebarSwipe and TextMagnifier ($t_{5.82}=-14.23$, p<0.0001), and TapFix and TextMagnifier ($t_{5.39}=-21.82$, p<0.0001). Bonferroni correction was applied to adjust for multiple comparisons.

Examining task completion time by its individual components shows that TapFix corrections on average happen within the time the user activates either of the baseline methods and positions the cursor. While cursor positioning with TextMagnifier is faster, it is hindered by a long activation time (almost double that of the other two methods), whereas SpacebarSwipe suffers from the opposite phenomenon. The cursor positioning time will be further investigated and discussed in Section 5.3.

The mean of the insertion time component was 0.27 s (*SD* = 0.62) for TapFix, 0.53 s (*SD* = 0.55) for SpacebarSwipe, and 0.55 s (*SD* = 0.58) for TextMagnifier. For the deletion time component, the means were 0.38 s (*SD* = 0.37) for TapFix, 0.61 s (*SD* = 0.60) for SpacebarSwipe, and 0.61 s (*SD* = 0.60) for TextMagnifier.

Due to a non-normal distribution of the values, significance testing used a Friedman test. The effect of the correction method on both the insertion time ($\chi^{2}=22.0,\;p\;<\;0.0001,\;\mathrm{df}=2$) and the deletion time component was statistically significant ($\chi^{2}=16.93,\;p\;<\;0.001,\;\mathrm{df}=2$). Pairwise post hoc comparisons using Conover’s *F*-test revealed that this difference was only statistically significant between pairs of TapFix and the baseline methods, but not between the baseline methods themselves. This was expected, as both baseline methods are similar in the distance the finger travels: Either from the space bar just below the keyboard, or form the text field just above. Additionally, the insertion and deletion components for TapFix were expected to be lower as well, as Swap-type tasks essentially have a zero insertion and deletion times for this method, and the data are averaged over all correction types.

#### 5.2.2. Impact of Target Word Length

**Figure 12:**
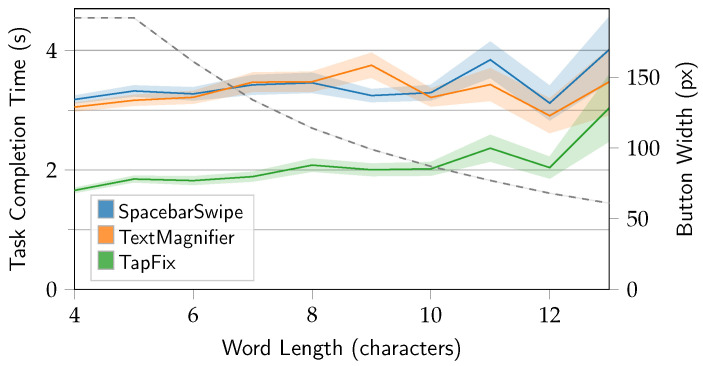
Task completion time (s) by target word length. Envelope shows ±1 *SE*. Dashed line and right-side axis represent TapFix character button width.

Figure 12 shows the task completion time in relation to the length of the word corrected.

The target word length was not a controlled variable, and target words for typing mistake induction and subsequent correction were randomly picked as described in Section 4. Due to random sampling and the distribution of words in the underlying phrases dataset, the number of samples at the tail end of the graph is limited, and the standard error is higher. For example, there are only 19 samples for word length 13 and 55 for length 12, respectively. Generally, it can be said that the task completion time seems to grow with the target word length for all three methods. Regarding TapFix, the shrinking character width is a non-linear relationship with the task completion time. Even at a target word length of 12, when TapFix characters were 32 px narrower than keys on the keyboard, the time to complete the task was not significantly affected. However, whether this effect was statistically significant cannot be conclusively answered from the data due to the aforementioned low sample size at the tail end of the distribution.

To put the impact of target word length into context, an analysis of word frequency and size was conducted based on the British National Corpus [30], p. 334. The analysis revealed that only 2.95% of words (or 0.22%, adjusted for frequency) are longer than 13 characters. Conversely, 90.9% of the words are 8 characters or less, with a weighted mean of 4.59 characters. Only 5 of 64,588 words (0.074%) in the corpus are longer than 21 characters and would thus exceed the limit of characters TapFix displays without overlap.

### 5.3. Cursor Positioning Time

The grand mean for cursor position time was 1.54 s. Note that the correction type is irrelevant for the cursor positioning time (see Section 4.4).

**Figure 13:**
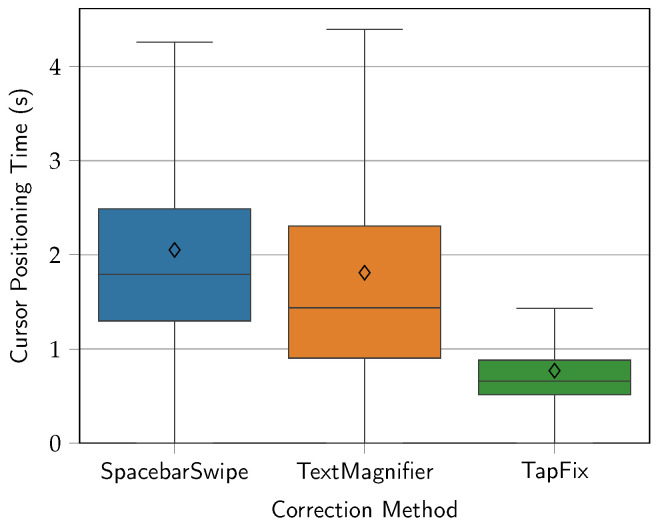
Cursor positioning time (s) by correction method. Diamonds show the mean times.

The mean times per correction method were 2.05 s for SpacebarSwipe (*SD* = 1.41), 1.75 s for TextMagnifier (*SD* = 1.53), and 0.95 s for TapFix (*SD* = 0.51). As such, TextMagnifier trials were 13.4% faster than SpacebarSwipe trials, and TapFix trials were 135.4% faster than TextMagnifier and 166.9% faster than SpacebarSwipe trials. The distribution of the results, as well as minimum and maximum values, are seen in Figure 13. Note that once again, data points above the upper fence are not shown in the figure. All methods exhibit a lower bound of the cursor positioning time very close to zero. There are two reasons for this: When the erroneous character is located at the end of a word, positioning with the SpacebarSwipe or TextMagnifier method is in most cases almost immediate, as iOS allows the user to directly place the cursor at the end (or start) of a word with a single tap, though not in between characters within a word. Likewise, for TapFix tasks of correction type Insert, a missing character in the middle of a word results in a zero positioning time as well, since no further movement of the newly inserted character is needed.

A one-way repeated-measures ANOVA was conducted to compare the effect of the correction method on the cursor positioning time. Shapiro–Wilk tests and a manual inspection of the Q-Q plot confirmed a satisfactory level of normality. Mauchly’s test indicated that the assumption of sphericity had not been violated ($\chi^{2}=0.72$, p=0.70). The effect of the correction method on the cursor positioning time was statistically significant ($F_{2,28}=109.6$, p<0.0001), and the effect size indicated a large effect ($\eta^{2}=0.67$). A post hoc analysis using pairwise *t*-tests revealed that the cursor positioning time was significantly different between SpacebarSwipe and TapFix ($t_{118}=11.0$, p<0.0001) and TapFix and TextMagnifier ($t_{118}=-8.61$, p<0.0001), but not between SpacebarSwipe and TextMagnifier ($t_{118}=1.90$, p>0.05). Once again, Bonferroni correction was applied to adjust for multiple comparisons.

Despite not being much slower on average than TextMagnifier, all except three participants voiced frustration with SpacebarSwipe. As already noted in the introduction to Section 5, this method’s mode of activation commonly troubled participants. Since moving their finger prematurely would cancel the activation, participants had to pay close attention to the screen and make sure the keyboard was fully greyed out before attempting to position the cursor. Furthermore, moving the finger on the space bar did not directly correspond to cursor movement, as the space bar does not span the entire width of the screen. One participant in particular pinpointed cursor acceleration as their main issue with this method: The cursor overshot and sometimes required multiple back-and-forth movements before eventually leveling out at the desired position.

On the other hand, only a few participants mentioned similar positioning issues with TextMagnifier. Of the three participants who did so, one said his thumb sometimes blocked his view, while two others felt more precision was needed when “dropping” the cursor to land at the intended position. Generally, participants seemed to prefer the direct translation of finger to cursor movement in this method, the usability implications of which are discussed in Section 5.4.

TapFix, as previously discussed, had much quicker positioning times, mainly due to the direct access to individual characters. This is also reflected in the lower spread of values around the median, which may indicate a higher level of robustness.

### 5.4. System Usability Scale

**Figure 14:**
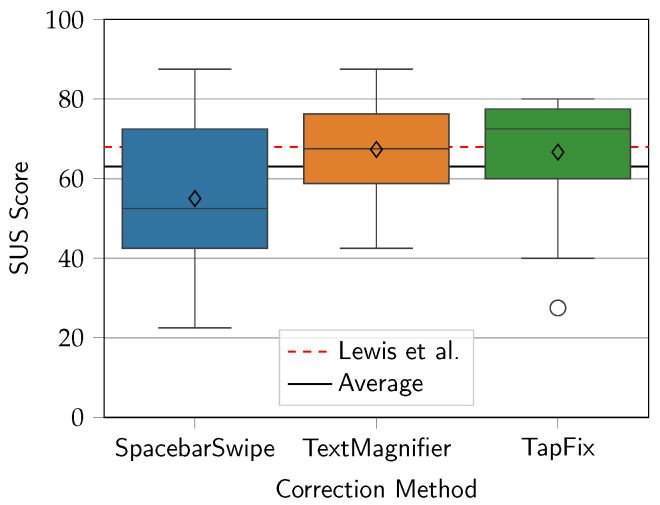
SUS scores of the three correction methods. Diamonds show the mean scores. Red dashed line from Lewis et al. [31].

The SUS scores of the corrections methods were calculated according to Brooke [24] and are shown in Figure 14. TextMagnifier achieved the highest mean usability score at 67.3, followed by TapFix with a score of 66.7 (−0.99%), and lastly TextMagnifier with a score of 55 (−18.3%). The differences were not statistically significant, as confirmed in a Friedman non-parametric test ($\chi^{2}$ = 1.66, *p* = 0.437, *df* = 2).

**Figure 15:**
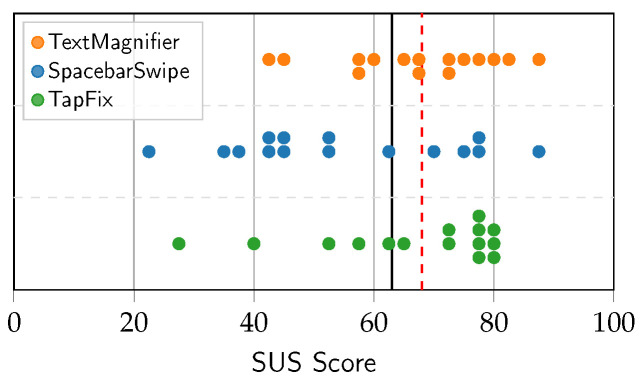
Swarm plot of SUS scores. Each marker is the response for one participant.

Comparing the SUS scores to the benchmark average of 68—as articulated by Lewis et al. [31]—the scores are slightly below average for TextMagnifier and TapFix and far below average for SpacebarSwipe. Interestingly, despite the mean SUS score for TapFix falling below the benchmark, the median at 72.5 was above average, whereas for the other two methods the medians (52.5 for SpacebarSwipe, 67.5 for TextMagnifier) were similar to their means. Closer inspection of Figure 15 shows nine measurements (60%) grouped closely together within the range of 70 to 80. That the majority of participants provided these relatively high scores possibly indicates a polarized response to the TapFix method. Indeed, the participants providing the two lowest scores for the TapFix method commented they either felt the triple-tap activation was unintuitive, or that TapFix hindered their correction speed, despite achieving objectively faster speeds in comparison to the other methods.

These findings bear on the inherently different correction approach using TapFix: While the cursorless approach appealed to a large subset of the participants, others may have been put off by the unconventionality of the method. On the other hand, the more uniform spread of scores for the SpacebarSwipe and TextMagnifier methods suggests that these were perceived more consistently among all subjects.

### 5.5. Task Load Index

**Figure 16:**
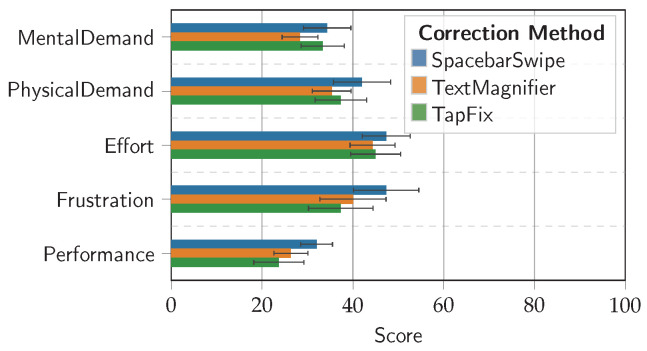
NASA-TLX sub-scores by correction method. Lower scores are better. Error bars show ±1 *SE*.

The grand mean of the NASA-TLX workload scores overall correction tasks was 36.9 (*SD* = 21.77). The means for the individual sub-scores were 45.6 (*SD* = 19.9) for Effort, 41.6 (*SD* = 27.6) for Frustration, 32.0 (*SD* = 17.9) for Mental Demand, 38.2 (*SD* = 20.9) for Physical Demand, and 27.3 (*SD* = 16.8) for Performance. Bear in mind that “Performance” in the NASA-TLX is qualitative (*How successful were you in accomplishing what you were asked to do?*). See Figure 16. A Friedman test revealed significant differences only for the Performance sub-scale ($\chi^{2}=6.30,\;p<0.05,\;\mathrm{df}=2$). Post hoc pairwise comparisons using Conover’s *F*-test revealed a significant difference between TapFix and SpacebarSwipe (|2.4−1.5|>0.651, df=28). Differences between the other two combinations were not statistically significant.

Yet, while there is not a statistically significant difference in most of the TLX dimensions between the correction methods, the data from the TLX questionnaire generally support the findings discussed earlier. For example, SpacebarSwipe yielded the highest frustration score of the three correction methods. This concurs with reports of participants struggling to activate this method, as previously discussed in the introduction to Section 5 and further described in Section 5.3. Additionally, the perceived better Performance score of TapFix compared to SpacebarSwipe is interesting due to the unfamiliarity of the participants with both methods prior to the experiment: All except two, and especially the participants who owned iOS-based devices, were surprised to learn that the SpacebarSwipe method existed. Out of the two who had previous experiences with it, only one indicated it as their preferred method to correct typing mistakes. As participants rated their perceived performance with TapFix on par with the familiar TextMagnifier, this suggests a high degree of adaptability for TapFix. Furthermore, the statistically significant performance advantage for TapFix over SpacebarSwipe in both perception (qualitative) as well as measurements (quantitative), as noted in Section 5.2, underlines the method’s effectiveness in typing mistake correction compared to the baseline methods.

### 5.6. Limitations

While great care was taken to ensure high overall validity of the experiment presented in this work, a few limiting factors remain. These are examined in this section.

#### 5.6.1. Choice of Baseline Methods

In this experiment, TapFix was compared against two baseline methods, TextMagnifier and SpacebarSwipe, which are integrated into and available on devices running Apple iOS. No other methods (e.g., from Related Work, Section 2) were taken into account in this comparison. The reasons were two-fold: First, TapFix is not a replacement for Autocorrect: All related work cited seeks to improve existing correction methods by making Autocorrect either easier to apply or more powerful. However, this also makes the methods inapplicable to this experiment, as the use-case for TapFix and the two baseline methods is when this automatic correction fails, forcing the user to manually correct the mistake. Potential causes for this situation are plentiful: The participants in the experiment mentioned deliberate misspelling of words, foreign names and words (as discussed in Section 1), as well as Internet slang terms, many of which are influenced by or directly loaned from African-American vernacular English. Second, all related work operated on (now) older versions of Android, and porting the individual methods to iOS would be tedious and in most cases futile, due to the aforementioned basis for comparisons in this experiment. This forms a limitation herein, as further cursor positioning or other unconventional approaches to compare the TapFix method against would be desirable.

#### 5.6.2. Focus on Single-Letter Corrections

The focus within all correction tasks was on single-letter corrections, with the exception of the Swap correction type: Swap is a special case, as it requires two separate insertions and deletions for the baseline methods, while allowing for correction with a single drag-and-drop action for TapFix (see the discussion in Section 5.2). In general, performing sequences of corrections might yield different results from the ones presented in this section, and thus forms a limitation of this work. However, comparison of the Swap-type task results for the baseline methods with the Replace results for TapFix allows for a projection of the results onto the special case of corrections of two adjacent typing mistakes. In particular, whether adjacent or not, this is equivalent to performing two separate Replace type actions with TapFix. By the results in Figure 10, this on average takes 2×1.98 s=3.96 s, and thus is still marginally faster than the baseline methods at a mean correction time of 4.3 s between both of them. This is an unrealistic estimate for TapFix, as it accounts for the method activation time twice, so actual measurements would be quicker.

Furthermore, cases where errors are not adjacent incur a speed penalty for the baseline methods to either reactivate the method to reposition the cursor, or delete and retype the characters between the two errors. At an average adjusted typing speed of 4.05 cps, there is an argument that in those cases, as well as sequences of more than two errors in general, completely deleting and retyping the word is faster than attempting to correct the individual mistakes. While this was tested, it is an interesting metric to analyze in the future.

#### 5.6.3. Effect of Word Length

The target words in the correction tasks were randomly chosen. As such, the target word length was uncontrolled and followed the distribution of the phrases dataset, which in turn is highly similar to that of the BNC corpus investigated in Section 5.2.2. Although improving external validty, this is somewhat limiting, as the data do not allow for conclusive statistical analysis of the effect of word length on correction speed.

#### 5.6.4. Limitations of the TapFix Method

One limitation of TapFix is word length. While it is possible to work with words of more than 21 characters, characters will start to overlap. This may be noticeable on phones with smaller screens. A potential option to address this is to allow word-wrapping—splitting longer words onto multiple lines. However, this would be tricky to implement, as gesture conflicts (such as between drag-and-drop and the Delete gesture) would have to be resolved somehow.

Word length is likely a minor issue, however, as words greater than 21 characters in length occur with frequency less than 0.1% in English, as detailed in Section 5.2.2.

## 6. Conclusions

This experiment compared TapFix against the two traditional text correction methods for touch-sensor displays, SpacebarSwipe and TextMagnifier. Correction tasks with TapFix were in most cases completed faster than using the traditional methods. In terms of speed, TapFix performed on average 44.1% faster than SpacebarSwipe and 43.0% faster than the TextMagnifier method. The differences were statistically significant for all correction task types, except for Insert-type tasks wherein the methods exhibited equal correction speed. While the difference in scores in the post-experiment System Usability Scale questionnaire was not statistically significant between the methods, closer inspection revealed a potentially polarized response of the participants to the TapFix method. The higher performance of TapFix was also reflected in user perception as shown by the NASA Task Load Index questionnaire. It revealed a statistically significant difference in perceived performance between TapFix and SpacebarSwipe, both of which were largely unknown to the participants prior to the experiment.

Future contributions might include testing TapFix in other environments and correction task types. An example is corrections in multi-line text fields, which are even more affected by occlusion, or corrections spanning more than a single character. Furthermore, testing with different populations (e.g., older people) might yield significantly different results. Lastly, an ethnographic study may be considered to further understand and address user text correction habits and situations where manual typing mistake corrections are required.

## Figures and Tables

**Table 1:** Related cursorless text correction research papers. *N* refers to the number of participants.

Authors	Year	*N*	Accuracy	Edit Time	Results	Notes
Zhang, et al. [21]	2019	20	88–97%	4.3 s–5.9 s	The proposed methods were on average −3.4% slower (Drag-n-Drop) to 32.5% faster (MagicKey) than the cursor-based method.	Typos, word-changes and replacements on a near, medium and far scale. Two of the three proposed methods were Neural Network assisted.
Cui, et al. [22]	2020	16	96–97%	5.62 s–10.22 s	The proposed method was 12.8% faster than the cursor-based method and 9.7% faster than the fastest two methods from Zhang et al. [21].	Improved method by removing manual selection and cursor placement operations from MagicKey by Zhang et al. [21].
Li, et al. [23]	2020	18	n/a	6.44 s	17% faster average action time for the proposed Swap method compared to the conventional cursor-based one.	Pre-study of user text correction habits and proposal of a method similar to MagicKey by Zhang et al. [21].

**Table 2:** Pairwise *t*-tests for task completion time. (Note: A and B reference the correction methods: SBS = SpacebarSwipe, TM = TextMagnifier, TF = TapFix; Sig.: +++ = *p* < 0.005).

Type	A	B	*t*	*df*	$p_{\mathrm{unc}}$	$p_{\mathrm{corr}}$	Sig.
Delete	SBS	TF	9.68	28	0.00	0.00	+++
Delete	SBS	TM	−0.00	28	1.00	1.00	–
Delete	TF	TM	−6.64	28	0.00	0.00	+++
Insert	SBS	TF	1.70	28	0.10	1.00	–
Insert	SBS	TM	0.26	28	0.80	1.00	–
Insert	TF	TM	−1.59	28	0.12	1.00	–
Replace	SBS	TF	7.55	28	0.00	0.00	+++
Replace	SBS	TM	0.47	28	0.64	1.00	–
Replace	TF	TM	−6.38	28	0.00	0.00	+++
Swap	SBS	TF	7.53	28	0.00	0.00	+++
Swap	SBS	TM	0.25	28	0.81	1.00	–
Swap	TF	TM	−8.58	28	0.00	0.00	+++

## Data Availability

Data are contained within the article.

## Appendix A

### Appendix A.1. Questionnaires

This appendix contains the questionnaires administered as part of the user study. The SUS questions were modified from the original SUS questions [24] to focus on an interaction method rather than an entire system.

#### Appendix A.1.1. Modified SUS

Users were asked to answer the following questions on an ordinal scale from 1 (*strongly disagree*) to 5 (*strongly agree*). For questions 1, 3, 7, and 8, higher scores are better. Questions 2, 4, 5, 6, 9, and 10 use reverse scoring with lower scores preferred. A score out of 100 is obtained by (SUM(Q1,Q3,Q7,Q8)-4+30-SUM(Q2,Q4,Q5,Q6,Q9,Q10))*2.5.

Q1. I think that I would like to use this method frequently.………………………
Q2. I found this method unnecessarily complex.………………………
Q3. I thought this method was easy to use.………………………
Q4. I think that I would need the help of a technical person to be able to (better) use this method.………………………………
Q5. I thought there was too much inconsistency in this method.………………………
Q6. I found this method very cumbersome to use.………………………
Q7. I would imagine that most people would learn to use this method very quickly.………………………
Q8. I felt very confident correcting typing errors using this method.………………………
Q9. It took me a while to learn how to properly use this method.………………………
Q10. I think that less technically inclined users would struggle with this method.………………………

The original questions were modified to refer to an interaction technique, rather than a “system”. The fifth question in the original questionnaire was deemed not applicable and was replaced by Q10.

#### Appendix A.1.2. NASA-TLX

Users were asked to answer the following questions on an ordinal scale from 1 (*very low*) to 20 (*very high*), except for question 3 on performance where the scale was from 1 (*perfect*) to 20 (*failure*). For all questions, lower scores are better. Note that the original question 3 on temporal demand was omitted.

Q1. How mentally demanding was the task?……………………………………………
Q2. How physically demanding was the task?……………………………………………
Q3. How successful were you in accomplishing what you were asked to do?………………………
Q4. How hard did you have to work to accomplish your level of performance?………………………
Q5. How insecure, discouraged, irritated, stressed, and annoyed were you?………………………

### Appendix A.2. Typo Generation

The following algorithm describes how typos were induced in target sentences from the MacKenzie and Soukoreff phrase set [27].

**Algorithm A1** Induce a Single Typo in a Target Sentence
1: **procedure** GenerateTypoSentence(S,T) ▹ Input sentence *S*, typo type *T*. 2: S←Lowercase(S) 3: W←ExtractWords(*S*) ▹ Ignore punctuation and whitespace. 4: **repeat** 5: i← Random index in [0,|W|−1] 6: **until** |W[i]|>3 ▹ Select a word of length at least 4. 7: w←W[i] 8: **switch** *T* **do** 9: **case** Replace 10: I←RandomIndices(*w*, *typoCount*) 11: **for all** j∈I **do** 12: $r_{j}\leftarrow$ Random character ∈{a,…,z} such that $r_{j}\neq w\left[j\right]$ 13: **end for** 14: $w^{\prime }\leftarrow$ReplaceCharacters($w,\;I,\;\{r_{j}\}$) 15: **case** Delete 16: I←RandomIndices(*w*, *typoCount*) 17: $w^{\prime }\leftarrow w$ 18: **for all** each j∈I in descending order **do** 19: N←Neighbours(w,j) ▹ Obtain characters adjacent to position *j*. 20: c← Random character such that c∉N 21: $w^{\prime }\leftarrow$InsertCharacter($w^{\prime },\;c,\;j$) 22: **end for** 23: **case** Insert 24: j← Random index in [0,|w|−1] 25: $w^{\prime }\leftarrow$RemoveCharacter(w,j) ▹ Remove character at index *j*. 26: **case** Swap 27: j← Random index in [0,|w|−2] 28: d← Randomly choose from {−1,+1} 29: k←j+d 30: **while** w[j]=w[k] **do** 31: Choose a new *k* adjacent to *j* ▹ Ensure characters are not the same. 32: **end while** 33: $w^{\prime }\leftarrow$SwapCharacters(w,j,k) 34: $W\left[i\right]\leftarrow w^{\prime }$ 35: $S^{\prime }\leftarrow$ReassembleSentence(*W*) 36: **return** $S^{\prime }$ 37: **end procedure**

**Function Descriptions:**

- ExtractWords—Splits the sentence into words while removing punctuation.
- RandomIndices—Returns a set of unique random indices within the chosen word; if the requested number exceeds the word length, defaults to one index.
- ReplaceCharacters—Substitutes the characters at the given indices with provided replacement characters.
- Neighbours—Returns the set of characters immediately preceding and succeeding the specified index.
- InsertCharacter—Inserts a character at a specified index (or appends if the index is at the end).
- RemoveCharacter—Removes the character at the specified index.
- SwapCharacters—Swaps the characters at two specified indices.
- ReassembleSentence—Reconstructs the full sentence from the list of words.
